# Our Summer Resorts

**Published:** 1874-07

**Authors:** Ben. H. Pratt


### THE BISTOURY.

Vol. X. ELMIRA, N. Y., JULY, 1874. No. 2.

### Contributions.

### OUR SUMMER RESORTS.

**Who Go There, and Why They Go.**

BEN. H. PRATT, A. M.

Every man and every woman in the middle and higher ranks of society, deems it essential, in these latter days, to spend from one week to three months of the year at some sort of a Summer Resort. Such a mania has this annual hegira assumed that it has effected most singular results, not only in the social status of the entire country, but also in the business of the nation, affecting alike its commerce, its science, its art, its literature, its humanities, its morals and its religion.

Time was when men and wom n stayed at home all summer and were content. Our grandsires—nay even our sires and their circumspect dames—deemed it no hardship to spend the entire year within their own domicils, caring, and cared for by, their growing families, and rearing them into healthful and heartwhole manhood and womanhood with no thought of that which we have come to believe a *sine qua non* in the bringing up of children—an annual trip to the springs, or the mountains, or the sea side.

The aforetime occasional has become the present universal. The Crœsus of the town, in the former days, may have taken a whim for the nonce and trundled his wife and family off for a fortnight's ramble among the wilds of an adjacent county, just for fun, and because he was at a loss what to do with himself. The confirmed invalid, upon whom his doctors had exhausted their skill and prescriptions, was experimented upon by a send-off to other climes and urged to divert his mind from himself by being thrown among scenes that were new and strange. The multitudes still pursued their business avocations—closed the shutters and kept the home cool in August—sprinkled the store, or office, or shop floor—sweat a little and puffed some—but never dreamed of seeking comfort or relaxation by plunging into the whirl of a fashionable summer resort and closing the house for the summer. The latter notion is a modern one, and the very fact that it has not arisen from a necessity, gives rise to a great horde of excuses for its universal adoption.

Health's demands are urged as rendering the spending of the summer abroad as essential. Recreation is also a grand theme upon which they enlarge who can't trump up an ailment. Comfort during the hot weather, is offered in lieu of other reason. The honest ones admit that they go to be in the fashion. It is very vulgar to stay at home all summer, say they, and we must go to keep up our standing in society.

The minister announces his annual bronchitis about the middle of July and the dear flock pack his portmanteau for him and donate him a good substantial check, and off he goes, leaving a relieved fold without a shepherd.

The Judge on the bench expedites the business of the early summer term, and gives listless heed to the promptings of the blind goddess, for his wife and daughters have their Saratogas all packed for the anxious rising of the court.

The Practitioner at the Bar is more interested in Long Branch vs. Cape May than in Smith vs. Brown, and his briefs are such in nature as well as in name, for he remembers last summer's *otium cum dignitate* on the beach, and is bent on an *ut supra.*

The Doctor is careful to withhold reducing precriptions from his patients, and gets them all into a capital tone by the end of June, so that he can then slip off for a month without anxiety as to the result of his last experiment.

The merchant balances his books and finds a fair cash margin, providing Skin, Flint & Gouge don't draw at sight, and he, too, takes train and a handful of railway checks for Dashaway Beach, where the salt air lures him into forgetfulness of panicky risks and a lot of green clerks at home.

Salesmen, having denied themselves the habit, for the past three months, of living beyond their salaries, pocket the balance due, and with what means they can secure from their uncle, and with what credit they can get at the tailor's, bloom out into bipedal butterflies and putting on millionaire style buy a lonely ticket for the Springs, musing upon the flirtationary programme which awaits them.

Chawles Augustus, son of Grip Scrouge, the fat banker, bids his man put all his new pantaloons and things into his twunk and bwing awound the dwag and twundle him to the daypo for the Sawatogah twain, foh the heat is so demmed oppwessive that he cawn't possibly enduah the woasting metwopolis any longah—positively cawn't; and leaves orders for ma to addwess him at Long Bwanch, care of his vewy pawticulah fwiend, Munchausen Shallowpate, Bawonet. Ta-ta, Ma!

Arabella, Araminta, Marie and Livonia have completed their one hundredth new dress apiece, with the help of ten or a dozen fashionable milliners, whose horrid presence in the house is about to be dispensed with for a season, and the dear creatures are all in a quiver over the low necked and swiss muslin and moire antique time they will have at their favorite resort, the very thoughts of which set them in a premonitory rhapsody.

The Hon. P. St. Roque, who was Paddy O'Rorke before the war and while he was making his first $100,000 in the sutler business along with the army of the Potomac, and who has grown great in the waist since his last job of furnishing spittoons for a certain famous court-house—he, with his obese wife and marketable daughters would as soon think of again removing from Fifth Avenue into Rag Alley, whence they sprang, as to pass the "hated terrum in the dusthy methropolus." "Uv coorse, Biddy"—he sometimes forgets himself and talks to his wife in this way—"we'll be afther waitin' to see where the Prisidint is to be, an' thin we'll orther our lando to be riddy upon our arrival, so we can dhroive to the 'otel in foine stoile."

What a sickly nation we have become, to be sure. We get along very well in the winter, and keep up pretty vigorously during the spring of the year, and until June one would scarcely believe there were so many seeds of disease latent in the great society element of the country. June, according to watering place statistics, is the invalid-making month of the year. By the first of July it is an unusual thing to find a healthy human being, except in the very lowest ranks of society. From the bronchial affection of the petted gentleman known as our devoted pastor, whose weekly oracular pulpitisms are rewarded by the mere pittance of ten thousand a year, and who is ambitiously committing annual martyrdom for the dear souls committed to his charge—from him to the tender lamb of the flock who is pimply, and must needs drink of the healing waters clad in a low-necked dress and tight corsets—there are all grades and phases of summer maladies. There is no end of rheumatisms, scrofulas, outcropping evidences of heart disease, cerebral difficulties, spinal disorders, pneumonic affections, liver complaints, scrofulous tendencies, general declines, want of tone, anæmias, systemic irregularities, dyspepsias, indispositions innumerable, all of which are beyond medical skill, and must be watered, or aired, or salted down at a Resort. Then there's the great army of run-down people, tuckered out folks, clean gones, overworked wretches, general debility cases, nerve shattered pitiables, hypochondriacs, all of whom give out utterly by the middle of June, and will die very quickly after the first of July unless they go to a watering place.

The man who invented Summer Resorts should have a monument erected to his memory in each and every one of our great omnium gatherums on the continent; for is he not the means of annually saving millions of his fellows? Behold how effectual the plan. Scarcely are our million invalids set down at their favorite Resort than they begin forthwith to mend. The first sniff of the magic atmosphere brings relief—the first meal recalls the lost appetite—the first ball reveals a renewed strength—the first evening upon the veranda proves that the affections have again asserted their wonted sway—the blanched cheek blooms—the dragging step quickens—

the dull eye brightens—the listless arms that erst found it tiresome to do up one's back hair, are now endowed with a clasping and embracing power that astonishes mamma and troubles papa. Truly, for the time, Esculapius is in the fix of Othello, his occupation's gone. No need of belladonna now for the glazing eye--no necessity for ferruginous concoctions to tone up the failing system—the livers all act, and the stomachs all digest—the rheumatisms have all fled—pimples disappear—hearts are all in a high state of flutter—spines straighten—dyspeptics become jolly—the tuckered obtain a new annual lease of vitality—nerves are lost sight of—hypochondriacs conclude to live on and forget the story of their ailments. Behold the miraculous power of healing that hovers over the fashionable resort!

Don't contradict us, please. Society is a unit upon this point. Society is on our side. We will not be responsible for your utter social annihilation if you assert that we are not absolutely correct in every particular above detailed. Remember that you are vulnerable and not immortal, ere you dare to brave the wrath of the great body social, and insinuate that we are a fool, and Society is a humbug, and watering places an abomination and a snare. Don't do it. Behold, is not Resort our summer god? Do we not fall down and worship him in the spirit? Do not we dress for him—lay our fortunes into his lap—offer up our daughters to him—pay our all, together with what we can borrow and steal, into his treasury? Do we not sacrifice business to him—build palatial hotels for him—annually create cities for him to dwell in—suffer extortion and all manner of imposition that he may smile upon us and command Society to fall upon her million knees and call us Honorable, or General, or Colonel, or the gifted, or the polished, or the greatest living Something or Other, from So and So?

Society has been revolutionized by the Resort. It is nothing if not migratory. The social metropolis is annually shifted. Mrs. Grundy goes to Newport, and in her train follow the creme de la creme of fashiondom. City shutters are closed and the front door knob, knocker and bell-pull grow dull, and servants hold high carnival in tapestried and brocaded saloons. No calls, no fetes, no receptions, no cards. Society has gone to the sea, to the spring, to the mountain, or in lieu thereof, if suffering from an attack of impecuniosity, is locked into a back room, up stairs, and "not at home" to anybody.

Business is conducted by upper clerks, who mope about and stalk through the empty salesrooms, moodily awaiting the senorial return, so that they too can join the revels of the summerlings at Dressington and exhibit their cassimers and patent-leathers. The glibness of the seller and the chaffing of the buyer are not exhibited over the counter, for the reign of commercial listlessness has set in, and Trade must grin and bear.

The Commercial Traveler is not seen skipping from step to curb, swinging his familiar portmanteau, for he has gone to curl his mustache and spank his legs with his dainty cane upon the verandah of the Ocean House, where employer and customer stroke their beards together, wondering how soon wife will be ready to go home.

Long haired and clean shaven Professors of every art, science and ism under the sun are there, and deserted study and studio and den in the hot city are gathering dust and mould to greet the September return of the master. The unfinished picture on the easel, the half completed bust on the pedestal, the dusty lens of the telescope in the observatory, the cabalistic diagrams on the desk, all tell the story of the annual scoot.

The September magazines run to emptings, for contributors have become demoralized with much lounging. Newspapers show a thin exhibit of editorial matter, for new hands are making eagle flights and goose descents while their chiefs are on the war trail to the summer capital, plotting the coming conflicts with hostile tribes or puffing the calumet of peace—three for a dollar—or taking notes of the council of "addition, division and silence," that hovers about the Great Father from the White Wigwam on the Potomac.

What is fashionable life? Three months of ball, fete, reception and gossip, in the metropolis—three months spent in selecting an excuse to go to a Summer Resort and getting something to wear thereat—three months of dressing, lounging, flirting, dancing, quizzing, envying, bathing, drinking, gaming, eloping, sweating, palpitating, dissembling and money spending—three months of gloating over and getting over it all—this is fashionable life in the nineteenth century.

